# Prevalence and correlates of post-traumatic stress disorder among survivors of road traffic accidents in Ethiopia

**DOI:** 10.1186/s13033-018-0229-8

**Published:** 2018-09-20

**Authors:** Kalkidan Yohannes, Abebaw Gebeyehu, Tewodros Adera, Getinet Ayano, Wubalem Fekadu

**Affiliations:** 1 College of Medicine and Health Sciences Department of Psychiatry, Dilla University, POBox 245, Dilla, Ethiopia; 2 Institute of Public Health, College of Medicine and Health Sciences, University of Gondar, Gondar, Ethiopia; 3 Research and Training Department, Amanuel Mental Specialized Hospital, Addis Ababa, Ethiopia; 4 College of Medicine and Health Sciences Department of Psychiatry, Bahirdar University, Bahidar, Ethiopia

**Keywords:** Post-traumatic stress disorder, Road traffic accident, Ethiopia

## Abstract

**Background:**

Post-traumatic stress disorder is the most common mental disorders occurring among survivors of road traffic accident. However, research into post-traumatic stress disorder and correlates in low and middle-income countries is limited. To the best of our knowledge, there is no published study of the post-traumatic stress disorder and associated factors conducted in Ethiopia. Therefore, this study aimed to determine the prevalence of post-traumatic stress disorder and associated factors among survivors of road traffic accident.

**Methods:**

Institution based cross-sectional study was conducted in May 2016. Data were collected using a pretested, structured, standardized post-traumatic stress disorder Checklist-Specific version (PCL-S) questionnaire. Systematic sampling technique was used to select the study participants. Binary logistic regression analysis was used to identify associated factors. Odds ratio with 95% CI was computed to assess the strength of associations.

**Results:**

The prevalence of post-traumatic stress disorder was found to be 22.8% (CI 19.2, 26.6) among survivors of road traffic accident. In the multivariable analysis, Being female [AOR = 2.23, 95% CI 1.40, 3.56], having poor social support [AOR = 2.1, 95% CI 1.34, 3.46], duration since accident (1–3 months) [AOR = 1.72, 95% CI 1.07, 2.76] and having depression [AOR = 3.46, 95% CI 1.99, 5.99] were significantly associated with PTSD among survivors of road traffic accident.

**Conclusion:**

In the current study the magnitude of post-traumatic stress disorder was high. Being female, poor social support, duration since the accident (1–3 months) and depression were found to be significant predictors of post-traumatic stress disorders. The finding suggests a need for early screening for post-traumatic disorder among survivors of road traffic accidents.

## Background

Road traffic accidents are a large and growing public health burden, especially in low income and middle-income countries [1–3]. According to the World Health Organization (WHO) (2015), globally, more than 1.2 million people die each year on the world’s roads, making road traffic accidents a leading cause of death globally [4]. According to the study in central Ethiopia between July 2007 and June 2012, there were 2335 RTAs registered in the eight police stations total of 515 people died in 389 (16.7%) of the accidents, while 549 (31.5%) and 681 (39%) people were severely and slightly injured, respectively. In addition, 316 (13.5%) and 290 (12.4%) of the crashes caused severe and slight injuries, respectively. The remaining collisions brought about only property damage [5].

Road traffic accidents (RTAs) can have serious and long-lasting consequences for survivors, both in terms of physical and psychological outcomes [6, 7]. The traumatic event such as RTA has a capacity to provoke fear, fear, helplessness, or horror in response to the treatment of injury or death [8].

According to DSM-IV, to be diagnosed with Post-Traumatic Stress Disorder (PTSD), a person must have re-experiencing symptoms (e.g. intrusive memories, nightmares), avoidance (e.g. avoiding reminders of the trauma) and hyperarousal (e.g. insomnia, startle response). Although it can begin right after a traumatic event, it is not diagnosed unless the symptoms last for at least 1 month, and either causes significant distress or interfere with work or home life [9]. The disorder may occur in people of any age who have been exposed to one or more exceptionally threatening or horrifying events [10]. In the adult population, the lifetime prevalence of PTSD found in community-based studies in approximately 8% [9].

Worldwide, PTSD is known as the most frequent mental disorder occurring in the aftermath of traumatic exposure [11]. It refers to deep emotional wounds results from exposure to an overwhelmingly stressful event, such as war, rape, accidents or physical abuse [12]. The epidemiologic research found that a considerable proportion of RTA survivors will develop psychological disorders following a RTA [13]. PTSD is the most common disorder following RTAs [14]. Prevalence rates of PTSD following a RTA vary from 6 to 45% depending on the study [15, 16]. Multiple factors can affect PTSD including being female, prior exposure to traumatic event, family and personal mental disorder history, lower education, low income, presence of comorbid mental disorder, trauma intensity, witnessing deaths that occurred during the RTA, lack of social support, unemployment after the event, persistent physical problem following the RTA and involvement in litigation/compensation [15, 17–21].

PTSD can have long-lasting consequences for recovery if left untreated, and it is associated with functional impairments and decreased health-related quality of life [21, 22]. Somatic symptoms were endorsed significantly more often by those who screened positive for PTSD than those who did not [23, 24]. PTSD generally increases absence from work [24]. The disorder is associated with substantial co-morbidity, such as depression and substance misuses, and significant economic burden [10].

Evidence indicated that post-traumatic stress disorder is the most common mental disorder occurring among survivors of road traffic accident [11]. It is also associated with personal suffering, morbidity, disability as well as poor quality of life [8, 10, 21, 22]. Despite this burden and consequences, there is a limited literature on the magnitude of PTSD and associated among survivors of road traffic accident in low and middle-income countries. Therefore, this study was intended to fill the gaps by assessing the prevalence and associated factors of PTSD among survivors of road traffic accident. It also helps to integrate mental health service in a primary healthcare unit by early diagnosis and timely treatment of comorbid cases.

## Methods

### Study setting and design

Institution based cross-sectional study was conducted at public hospitals in May 2016, Addis Ababa, Ethiopia. Of the 13 hospitals which are in Addis Ababa (capital city of Ethiopia), five hospitals which have organized orthopedic outpatient departments and wards were selected for our study. The included hospitals are Yekatit 12 hospital, Alert, Minilik II referral hospital, St. Paul millennium medical college branch—AaBETorthopedic, and Tikur Anbessa specialized hospital.

### Study population

The study population consisted of all adult survivors of RTAs who were on follow up at public hospitals in Addis Ababa who were included in the sample. Survivors of RTAs who were critically ill were excluded from the study.

### Sampling procedure

The sample size was determined based on single population proportion formula using Epi-info version 7 with a 95% CI, 5% margin of error and taking prevalence of post-traumatic stress disorder 13% from Kenya study [19]. Assuming a 10% non-response rate a total sample size of 531 persons who survived road traffic accident was required. Systematic sampling technique was used to select the study participants. Sampling interval was determined by dividing the total study population who had follow-up during four weeks data collection period by total sample size then the starting point was randomly selected.

### Data collection

Data were collected by trained psychiatry nurses. The questionnaire was pre-tested by taking 5% of the calculated sample size. The questionnaire contains socio-demographic characteristics (age, education, occupation, marital status and others). Clinical factors were collected by semi-structured questionnaires.

Data on the magnitude of posttraumatic stress disorder was collected through interview using standard PCL questionnaire. The post-traumatic stress disorder Checklist (PCL) come in three versions: the PCL-Military (PCL-M), PCL-Specific (PCL-S) and PCL-Civilian (PCL-C). Selected for this study is the PCLS, the PCL-S (specific) asks about symptoms in relation to an identified “stressful experience “The PCL-S aims to link symptom endorsements to a specified event. The PCL-S is a 17-item self-report measure reflecting DSM-IV symptoms of PTSD. Responses range from 1 to 5, and the total score is computed by summing all items. A total symptom severity score (range = 17–85) can be obtained by summing the scores from each of the 17 items that have response options ranging from 1 “Not at all” to 5 “Extremely”. The cut point is dependent on the population and use of the measure. When the estimated prevalence of PTSD will be on a range of 16–39% and the setting is specialized medical clinics (such as TBI or pain) a cut-off 36–44 on the PCL-S is a good predictor of a PTSD diagnosis. Higher scores indicate the probability of PTSD, while lower scores indicate no probability of having PTSD [25]. Nineteen studies examined total score internal consistency and all returned values above 0.75. Populations included Veteran samples, victims of interpersonal violence, among patients treated for breast cancer (0.87–0.92), patients with severe mental illness, and community adults [26, 27].

Social support was collected by Oslo 3-item social support scale, Oslo 3-item social support scale is 3-item questionnaire commonly used to assess’ social support and it has been used in several studies, the sum score scale ranging from 3 to 14, which has three broad categories: “poor support” 3–8, “moderate support” 9–11 and “strong support” 12–14 [28]. It is reliable in pretest (Cronbach’s α = 0.57) [26].

Depression was measured by using Patient Health Questionnaire-9 (PHQ-9) which is a 9-item depression screening and diagnostic questionnaire for MDD based on DSM-IV criteria with sensitivity 86% and specificity 67%. The PHQ-9 appears to be a reliable and valid instrument that may be used to diagnose MDD among Ethiopian adults [29].

### Data processing and analyses

Data were analyzed using SPSS version 20. Description of means, frequencies, proportions, and rates of the given data for each variable was calculated. Bivariate analysis was done to see the association of each independent variable with the outcome variable. Those variables having a p-value less than 0.2 were entered the multivariate logistic regression model to identify the effect of each independent variable with the outcome variables. A p-value of less than 0.05 was considered statistically significant, and an adjusted odds ratio with 95% CI was calculated to determine the association.

### Ethical consideration

Ethical clearance was obtained after approval from the Institutional Review Board (IRB) of the College of Medicine and Health Sciences, the University of Gondar and from Amanuel Mental Specialized Hospital. The data collectors have clearly explained the aims of the study to the study participants. Information was collected after obtaining written consent from each participant. In addition, we offered the participant the right to withdraw from the study at any time during the study period. Confidentiality was maintained throughout the study. Patients who were found to have post-traumatic stress disorder were referred for further investigations.

## Results

### Socio-economic and demographic characteristics

A total of 492 participants were included in the study, yielding a response rate of 92.65%. The mean ((± SD) age of the respondents was 30.12 (± 7.02) years. Among the respondents, 167 (33.9%) were in the age range of 26–35 years, 313 (63.6%) were male, 250 (50.8%) were married, 158 (32.1%) were attended secondary school, 204 (41.6%) were government and nongovernment employee, while 72 (14.6) were students. The median monthly income of the participants was 2000 ranges from 800 to 3000 ETB (Table 1).

**Table 1:** Distribution of road traffic accident survivors attending orthopedic unit of public hospitals, Addis Ababa, Ethiopia, 2016 (n = 492)

Characteristics	Frequency	Percentage
Sex		
Male	313	63.6
Female	179	36.4
Age (years)		
15–25	150	30.5
26–35	167	34
36–45	104	21.1
≥ 46	71	14.4
Marital status		
Single	207	42.1
Married	250	50.8
Separated	11	2.2
Divorced	13	2.7
Widowed	11	2.2
Educational status		
Unable to read and write	32	6.5
Primary school	148	30.1
Secondary school	158	32.1
Tertiary education	154	31.3
Occupation		
Government and non-Gov.t employee	204	41.5
Private business	136	27.6
Student	72	14.6
Daily labourer	55	11.2
Others^a^	25	5.1
Monthly income		
< 750 ETB	120	24.39
750–1250	63	12.81
> 1250	309	62.8

^a^ House wives and jobless

### The accident-related event, and clinical characteristics of the respondents

Regarding accident-related characteristics, the majority of the participant (367 (74.6%)) were admitted after the accident and treated by surgical intervention, 117 (23.8%) of the participant received compensation for the accident, and 48 (9.8%) of the respondents witnessed the death of someone during the same accident.

From the total study participants, about 82 (16.7%) had depression, 7 (1.4%) had history of mental illness, 22 (4.5%) had a family history of mental illness and 40 (8.1%) had co-morbid medical or surgical illness. Regarding site of injury, about 275 (55.9%) had lower limb injury and 163 (33.13%) had upper extremity injury. In addition, about 230 (46.7%) were interviewed within 1–3 months of their accidents (Table 2).

**Table 2:** Description of accident related and clinical factors among road traffic accident survivors attending orthopedic unit of public hospitals, Addis Ababa, Ethiopia, 2016 (n = 492)

Characteristics	Frequency	Percentage
Happing following accident (RTA)		
Admitted and surgery done	367	74.6
Admitted but no surgery done	85	17.3
Not admitted	40	8.1
Site of injury		
Upper limb injury		
Yes	163	33.13
No	329	66.87
Lower limb injury		
Yes	275	55.9
No	217	44.1
Trunk injury		
Yes	17	3.5
No	475	96.5
Spinal injury		
Yes	17	3.5
No	475	96.5
Head injury		
Yes	48	9.1
No	444	90.2
Litigation/compensation		
Yes	117	23.8
No	375	76.2
Family/family during accident		
Yes	78	15.85
No	414	84.14
Incidence during accident		
Yes	48	9.8
No	444	90.2
Ever treated for mental illness		
Yes	7	1.4
No	485	98.6
Depression		
Yes	82	16.7
No	410	83.3
Family member treated for mental illness		
Yes	22	4.5
No	470	95.5
Comorbid illness		
Yes	40	8.1
No	452	91.9
Duration since accident		
1–3 months	230	46.7
> 3 months	262	53.3

### Psychosocial and substance-related factors of the respondent’s

**Fig. 1:**
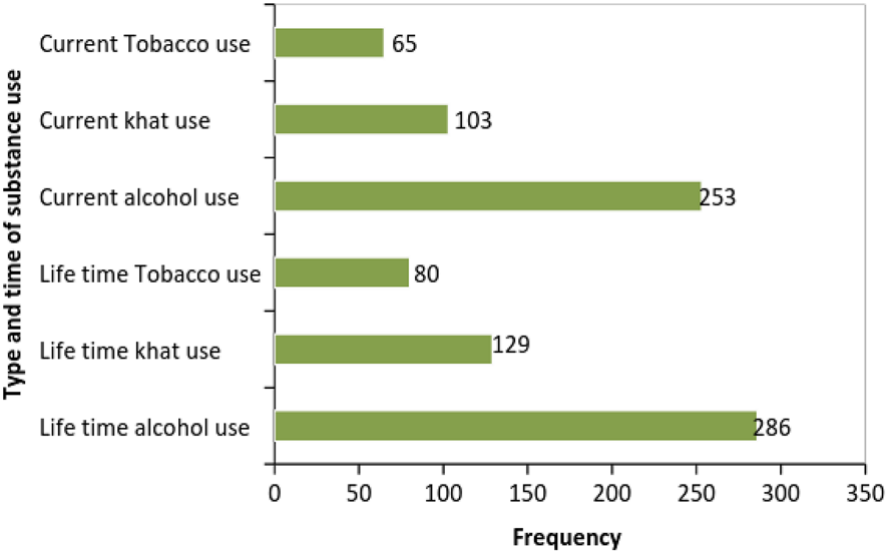
Substance related characteristics of road traffic accident survivors attending orthopedic unit of public hospitals, Addis Ababa, Ethiopia, 2016

About one-third of the participant with PTSD (190 (30.5%)) had poor social support, 208 (21.2%) had intermediate social support and 94 (10.6%) of the respondents had strong social support. Regarding current and lifetime substance use, more than half of the respondents (286 (58.1%) were lifetime alcohol users, and about half of the participants (253 (51.4%)) were current alcohol users (see Fig. 1).

### The prevalence of PTSD

In the current study, the magnitude of post-traumatic stress disorder was 22.8% with a CI of (19.2–26.6%). The prevalence rate was higher among females (32.4%) compared to males (17.25%). About 45.12% of the participants with PTSD had comorbid depression.

### Factors associated with PTSD

Multivariable logistic regression revealed the sex of the respondent, social support, duration since accident and depression had also shown significance. Females were 2.23 times more likely developed PTSD than males (AOR = 2.23, 95% CI 1.40, 3.56), those who had poor social support more likely developed PTSD than their counterparts (AOR = 2.1, 95% CI 1.34, 3.46), the odds of developing PTSD among those with depression were 3.46 times higher as compared to those without depression (AOR = 3.46, 95% CI 1.99, 5.99) and participants who had 1–3 months length time post-accident were 1.72 times more likely developed PTSD than respondents who experienced RTA more than 3 months of duration (AOR = 1.72, 95% CI 1.07, 2.76) (Table 3).

**Table 3:** Bivariate and multivariable analysis of factors associated with post-traumatic stress disorder among road traffic accident survivors attending orthopedic unit of public hospitals, Addis Ababa, Ethiopia, 2016 (n-492)

Characteristics	PTSD		COR (95% CI)	AOR (95 CI %)
	YES	NO		
Sex				
Male	54	259	1	
Female	58	121	2.29 (1.49, 3.53)	2.23 (1.40, 3.56)**
Age (years)				
< 20	21	41	2.15 (1.08, 4.29)	2.24 (0.86, 5.83)
20–29	43	123	1.54 (0.87, 2.69)	1.67 (0.86, 3.22)
30–39	22	115	0.80 (0.42, 1.52)	0.95 (0.47, 1.92)
40 and above	24	101	1	
Occupation				
Unemployed	36	96	1.40 (0.88, 2.21)	0.85 (0.47, 1.53)
Employed	76	284	1	
Marital status				
Married	48	202	1	
Single and previously Married	64	178	0.05 (0.98, 2.31)	1.25 (0.73, 2.14)
Happing following accident				
Hospitalization and surgery done	92	275	1.75 (1.03, 2.99)	1.7 (0.97, 3.32)
No-surgery done	20	105	1	
Social support				
Poor	58	132	2.01 (1.31, 3.09)	2.1 (1.34, 3.46)**
Good	54	248	1	
Lower extremity injury				
Yes	71	204	1.49 (0.96, 2.30)	1.16 (0.65, 2.08)
No	41	176	1	
Depression				
Yes	37	45	3.67 (2.22, 6.06)	3.46 (1.99, 5.99)***
No	75	335	1	
Duration since accident				
1–3 months	67	163	1.98 (1.29, 3.04)	1.72 (1.07, 2.76)*
> 3 months	45	217	1	
Upper extremity injury				
Yes	29	134	0.64 (0.40, 1.02)	0.6 (0.35, 1.21)
No	83	246	1	
Litigation/compensation				
Yes	20	97	2.36 (1.40, 3.96)	0.73 (0.40, 1.35)
No	92	283	1	

*p-value less than 0.05; **p-value less than 0.01; ***p-value less than 0.001

## Discussion

### The prevalence of PTSD

In this study, the prevalence of PTSD among survivors of RTAs and their possible associations with different variables were assessed. The findings from the current survey revealed a significant proportion of peoples who experienced RTA had PTSD. The prevalence of PTSD was found to be 22.8% (CI 19.2, 26.6) among survivors of RTA. The magnitude of this study was in line with the study done in Iran which was 19.2% [30], the finding also agreed with that of South Africans’ study who reported a PTSD prevalence of 22.9% at 1 month, about 19.6% percent at 3 months among trauma victims [31] and that of Nigerian study at 26.7% [32]. However, the finding of this study was lower than the Serbian study 36% [33]. The magnitude of PTSD among road traffic accident survivors vary widely as a result of the country of study, sample size, the time point of PTSD assessment and measure used to assess PTSD [15].

Contrarily, the finding of this study was higher compared to Kenya 13.3% [19] and study from Cape Town, revealed 12.2% of PTSD [31]. The possible explanation for the observed differences could be the difference in tool, which is Kenya’s study used both IES-R for screening and thereafter an experienced mental health clinician used the structured psychiatric interview (SPI) and the Diagnostic and Statistical Manual (DSM-IV) to make a diagnosis but the current study used only PCL-S which is a screening tool. In the case of South African’ study, the study was conducted 6-month of the post-accident period, but the current study assessed all RTA survivors from 1 month of the post-accident period.

### Factors associated with PTSD

This study revealed that factors associated with PTSD for RTA survivors are being female, poor social support, depression and time length post-accident [1–3] months. The odds of developing PTSD among females was two times (AOR = 2.23, 95% CI 1.40, 3.56) compared to males following RTA. This may be due to different ways of responding to danger and expressing distress in two sexes and females have found to be more likely than males to experience intense fear, horror, or helplessness in response to a traumatic event. The finding was supported by other studies from Nigeria [18]. Studies conducted in different areas on gender differences in PTSD after MVA revealed the risk of developing PTSD at 1 month was 4.39 times greater in women than men and other meta-analyses of sex differences in the development of PTSD support the current finding [17, 34, 35].

Social support was significantly associated with PTSD in which those participants with poor social support were more than two times (AOR = 2.1, 95% CI 1.34, 3.46) more likely to have PTSD respectively as compared to those with strong social support. This is maybe because not having strong social support after exposure to traumatic injury may lead to poor mental health since positive social support appears to mitigate the negative effects of traumatic injury and those who have poor social support may not develop proper coping strategies after trauma. The current finding is supported by a study from New York on social bonds and PTSD which explains social support as a robust predictor of PTSD [15, 20].

In addition, depression was significantly associated with the presence of PTSD in this study. Those patients with depression were more than three times (AOR = 3.46, 95% CI 1.99, 5.99) more likely to have PTSD as compared to their counterparts. This could be due to the fact of having another psychiatric diagnosis increase the risk of developing PTSD and poor long-term health outcomes including impaired physical functioning and lower self-reported quality of life may contribute. The current finding was supported by international studies from Cape Town [31] and Nigeria [18].

Furthermore, duration since accident, is found to be significantly associated with PTSD; the odds of developing PTSD among patients who were experienced road traffic accident 1 to 3 month before data collection time were 1.72 times (AOR = 1.72; 95%CI 1.02, 2.76) more likely to have PTSD as compared to patients who were injured before 3 months of data collection time. The reason could be the gradual decline over the course of the year. This finding was supported by studies from California [17] and South Africa [31].

However, there has been disagreement in the literature regarding whether or not marital status, site of injury, involvement in litigation/compensation predicts later PTSD [24, 36, 37], who reported a positive association between marital status, occupational status, and site of injury. The possible reason could be the difference in time of study and the possible methodological difference in conducting the studies.

### The strength and limitation of the study

The study has several strengths. First, the sample is large and from a well-defined catchment area. Second, we used the standardized instrument for measuring PTSD (post-traumatic stress checklist-specific version (PCL-S)).

A limitation of the study is that since the study assessed only the latest RTA that subjects were being treated for. Other different traumas that could have occurred in the subjects’ lifetime were not taken into account. These could have an influence on these results. Another limitation is due to the cross-sectional nature of the study of the association between different factors and PTSD does not imply causation.

## Conclusion

In the current study, the magnitude of PTSD was high. Female sex, having poor social support, having depression and duration since accident [1–3] months were significantly associated with PTSD. Orthopedic clinics should develop guidelines to screen and treat PTSD among survivors from a RTA. Further research on risk factors of PTSD should be conducted to strengthen and broaden the current findings.
